# Tau (297‐391) forms filaments that structurally mimic the core of paired helical filaments in Alzheimer’s disease brain

**DOI:** 10.1002/1873-3468.13675

**Published:** 2019-12-01

**Authors:** Youssra K. Al‐Hilaly, Bronwen E. Foster, Luca Biasetti, Liisa Lutter, Saskia J. Pollack, Janet E. Rickard, John M. D. Storey, Charles R. Harrington, Wei‐Feng Xue, Claude M. Wischik, Louise C. Serpell

**Affiliations:** ^1^ Dementia Research Group Sussex Neuroscience School of Life Sciences University of Sussex E Sussex UK; ^2^ Chemistry Department College of Science Mustansiriyah University Baghdad Iraq; ^3^ School of Biosciences University of Kent Canterbury UK; ^4^ Institute of Medical Sciences University of Aberdeen UK; ^5^ Department of Chemistry University of Aberdeen UK; ^6^ TauRx Therapeutics Ltd. Aberdeen UK

**Keywords:** Alzheimer’s disease, neurofibrillary tangles, paired helical filaments, tau

## Abstract

The constituent paired helical filaments (PHFs) in neurofibrillary tangles are insoluble intracellular deposits central to the development of Alzheimer’s disease (AD) and other tauopathies. Full‐length tau requires the addition of anionic cofactors such as heparin to enhance assembly. We have shown that a fragment from the proteolytically stable core of the PHF, tau 297‐391 known as ‘dGAE’, spontaneously forms cross‐β‐containing PHFs and straight filaments under physiological conditions. Here, we have analysed and compared the structures of the filaments formed by dGAE *in vitro* with those deposited in the brains of individuals diagnosed with AD. We show that dGAE forms PHFs that share a macromolecular structure similar to those found in brain tissue. Thus, dGAEs may serve as a model system for studying core domain assembly and for screening for inhibitors of tau aggregation.

## Graphical abstract

Truncated tau (297‐391) is the principal component of the core of paired helical filaments (PHFs) in neurofibrillary tangles isolated from Alzheimer’s brain tissue. Truncated tau forms filaments *in vitro* that share macromolecular characteristics with PHFs found *in vivo*. Truncated tau, without modification, is sufficient for the assembly of PHFs.

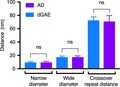

## Abbreviations

**AD**, Alzheimer’s disease

**AFM**, atomic force microscopy

**Cryo‐EM**, cryo‐electron microscopy

**NFTs**, neurofibrillary tangles

**PHFs**, Paired helical filaments

**SFs**, Straight filaments

**TEM**, transmission electron microscopy

The pathology of Alzheimer’s disease (AD) is characterised by the accumulation of amyloid‐β protein in senile plaques and tau in neurofibrillary tangles (NFTs). This is associated with the neurodegenerative features of the disease which includes oxidative stress, inflammation and compromised axonal transport, eventually resulting in neuronal cell death 1. Tau protein is a product of the *MAPT* gene, and alternative mRNA splicing generates six different isoforms of tau varying in their length. The C terminus contains three or four imperfect tandem repeats of a 31 or 32 amino acid sequence 2, 3, 4. Tau is a natively unfolded protein which plays an important role in microtubule assembly and stabilisation, vital for effective axonal transport and signal transduction 5. More recently, it has been identified in nucleolar functions where it has been seen to associate with nucleic acids 5.

In AD and other tauopathies, tau is deposited as paired helical filaments (PHFs) and straight filaments (SFs) in NFTs in cell bodies of neurons. The filaments share a cross‐β structure with amyloid fibrils 6, and recent cryo‐electron microscopy (cryo‐EM) studies have revealed atomic details of filaments isolated from brain tissues of patients with AD 7, Pick’s disease 8 and chronic traumatic encephalopathy 9. PHFs and SFs from AD are formed by two protofilaments containing parallel in‐register β‐sheet structures. The ordered structure resolved by cryo‐EM (residues 306‐378) consists of several β‐strands connected by β‐bends and a β‐helix conformation to form a single layer of the cross‐β structure. The specific conformations of the ordered domains of the filaments formed in the three conditions differ, but all encompass approximately residues 304‐380 of the 4‐repeat isoform or homologous residues of the 3‐repeat isoform. All share a similar consensus conformation comprising a hairpin structure, with different locations of poorly resolved cofactors and different modes of association between each protofilament. The ordered domains that have been resolved by cryo‐EM are all somewhat shorter than the fragment originally isolated from proteolytically stable PHF core preparations (residues 296‐391) 10.

Full‐length and fragments of tau have been studied extensively to ascertain how they form filaments. However, often these are made with the anionic cofactor heparin 11, 12, 13 RNA 14 or fatty acids 15, which have been thought to be necessary to initiate assembly. Recently, it has been found that the filaments prepared using anionic cofactors are structurally heterogeneous and distinct from those found in any of the diseases studied to date 16, 17. Therefore, the field is in need of a tractable and relevant form of tau for studying self‐assembly and pathology, and potentially developing therapeutic approaches targeting pathological aggregation of tau protein.

dGAE is a truncated form of tau protein comprising 95 amino acids that correspond to residues 297‐391 and encompasses repeat regions 3 and 4 of the 4‐repeat isoform. It corresponds to one of the species isolated from proteolytically stable AD PHF preparations 10. A fragment of tau ending at residue 391 has been identified in amorphous deposits in brain tissue sections 18 and within fuzzy‐PHFs that retain N‐ and C‐terminal domains of tau isolated from AD brain 19. The dGAE peptide has been suggested to represent an important stable core region of full‐length tau 19, 20, 21, 22, 23 and contains the ordered sequences that have been resolved by cryo‐EM in AD 7. We have previously shown that dGAE can self‐assemble into filaments without the addition of anionic cofactors and that these filaments not only share characteristics with amyloid, but also resemble the PHFs found in AD brains 24. The self‐assembled filaments were mainly PHFs with a minority (approximately 10%) of SFs (see Ref. 24). Here, we have performed an in‐depth study to compare the macromolecular structure of the PHFs in NFTs in the brain of an AD patient with the PHFs formed by dGAE. Using transmission electron microscopy (TEM) and atomic force microscopy (AFM), we show that these dGAE PHFs are comparable to PHFs found in AD brains at a macromolecular level.

## Materials and methods

### Brain tissue block preparation

Alzheimer’s disease brain tissue from middle frontal gyrus was obtained from London Neurodegenerative Diseases Brain Bank. Removal of the tissue was carried out according to Local Ethics Committee guidelines and informed consent for brain donation was obtained from the next of kin. The tissue was stored at −80 °C in a designated locked HTA freezer. Frozen tissue was transported on dry ice in order to preserve its structure and integrity to a Class II Type A2 biological safety cabinet. A sample of around 1 mm^3^ was excised using a scalpel blade (#22), placed immediately into a 1.5 mL Eppendorf tube containing a fixative solution (4% paraformaldehyde, 0.1% glutaraldehyde in PBS 1×) and left at 4 °C overnight. The following day, the fixative solution was washed out using PBS five times over a period of 4 h. During the washes, samples were rotated at 4 °C. This washing was necessary to remove residual aldehyde which can interfere with labelling in subsequent steps. Following the buffer rinse, samples were dehydrated using solutions of increasing EtOH concentrations (30%, 50%, 75%, 90%, 3× 100%, each for 20 min). Following the 100% EtOH step, the sample was prepared for embedding in resin (UNICRYL; BBI Solutions, Crumlin, UK) by primary incubation in a 100% EtOH : resin (2 : 1) solution for 2 h followed by a secondary incubation using a 1 : 2 ratio (30 min for both incubation steps) before moving into complete resin overnight at 4 °C. The following day the resin was replaced with fresh resin, and the sample was moved into a BEEM capsule (Agar Scientific, Essex, UK). Resin polymerisation was performed under UV light for 3–4 days at 4 °C until the resin was fully polymerised. The finished block was then removed from the BEEM capsule and prepared for ultrathin sectioning.

### Ultrathin sectioning

Polymerised tissue blocks were mounted on the sample holder of a Leica EM UC7 ultramicrotome. The tissue was located in the block and an area of roughly trapezoidal shape was cut using a razor blade (Astra superior platinum double edge razor blade). In order to remove the superficial layer of resin present on the tissue, a glass knife was used initially. Once the tissue was exposed, ultrathin sections of 60 nm were cut using a diamond knife (Ultra Diamond Knife – Wet 45^°^ 2.5 mm). Sections were collected on hexagonal 300‐mesh Nickel grids (3.05 mm; Agar Scientific) ready for immunogold labelling.

### Immunogold labelling of tissue sections for TEM

An established methodology was followed for the immunogold labelling 25. PBS+, a modified PBS at pH 8.2 containing 1% BSA, 500 μL·L^−1^ Tween‐20, 10 mm Na EDTA and 0.2 g·L^−1^ NaN_3,_ was used for all the following procedures, including dilutions of antibodies and secondary gold probes. PBS + buffer was used for immunoreagent dilutions and for rinsing. The grids were incubated first with normal goat serum (20 μL; 1 : 10 dilution) for 30 min at room temperature in order to block the binding of nonspecific secondary antibodies. Grids were then labelled with either T22 rabbit polyclonal anti‐tau antibody (ABN454; Merck Millipore, Darmstadt, Germany) (1 : 1000 dilution), total tau rabbit polyclonal anti‐tau antibody (SAB4501821; Sigma‐Aldrich Company Ltd., Gillingham, UK) (1 : 1000 dilution) or mAb 423 (1 : 20 dilution) 19, 26 and incubated overnight at 4 °C. The sections were rinsed 3 × 2 min with PBS + buffer. The sections were then immunolabelled either with goat anti‐rabbit IgG with 10‐nm gold particles (GaR10) or, for mAb 423, with goat anti‐mouse IgG with 10‐nm gold particles (GaM10) as secondary probes (all 1 : 10 dilution), for 1 h at room temperature. After 3 × 10‐min PBS + buffer and 4 × 5‐min distilled water rinses, the grids were then poststained with 0.22‐μm‐filtered 0.5% (w/v) aqueous uranyl acetate (positive stain) for 1 h. The grids were then rinsed a further 5 × 2 min with water before allowing to dry. TEM projection images were collected using a JEOL JEM1400‐Plus Transmission Electron Microscope operated at 120 kV equipped with a Gatan OneView camera (4k × 4k). Images were recorded at 25 fps with drift correction using GMS3. Only filaments with a twisted appearance were selected for analysis.

### Assembly of truncated dGAE tau fibrils

dGAE (300 μm) was incubated in phosphate buffer (10 mm, pH 7.4) containing 10 mm DTT at 37 °C with agitation at 400 oscillations per minute (Eppendorf Thermomixer C, Eppendorf, Germany) for 48 h.

### Negative stain TEM

Electron microscopy grids were prepared by placing a sample of dGAE fibrils (4 μL) onto formvar/carbon‐coated 400‐mesh copper grids (Agar Scientific). Excess sample was blotted with filter paper and then washed with 0.22‐μm‐filtered milli‐Q water (4 μL). Uranyl acetate (2% w/v; 4 μL) was added to the grid and left for 1 min before blotting. The grid was then allowed to air‐dry. TEM projection images were collected using a JEOL JEM1400‐Plus Transmission Electron Microscope operated at 120 kV equipped with a Gatan OneView camera (4k × 4k). Images were recorded at 25 fps with drift correction using GMS3. Only filaments with a twisted appearance were selected for analysis.

### Immunogold labelling of dGAE filaments for TEM

Briefly, the immunogold labelling was performed by placing 4 μL of a dGAE filament preparation for 1 min on Formvar/carbon‐coated 400‐mesh copper TEM support grids (Agar Scientific), and then the excess was removed using filter paper. For blocking, grids were incubated with normal goat serum (1 : 10 dilution) for 30 min at room temperature and then incubated with mAb 423 (1 : 20 dilution) for 2 h at room temperature. (mAb 423 was raised against PHFs from AD brains and detects tau protein C‐terminally truncated at Glu‐391 as reported in 19, 26). Grids were washed three times with PBS+ for 2 min each and then incubated with GaM10 secondary antibody (1 : 10 dilution) for 1 h at room temperature. The grids were washed five times with PBS+ for 2 min each followed by five washes with distilled water (0.22‐μm‐filtered) for 2 min each. The filaments were negatively stained with 2 % (w/v) uranyl acetate (0.22 μm filtered) for 1 min. TEM projection images were collected using a JEOL JEM1400‐Plus Transmission Electron Microscope operated at 100 kV equipped with a Gatan OneView camera (4k × 4k). Images were recorded at 25 fps with drift correction using GMS3.

### Atomic force microscopy

The dGAE fibril samples were prepared for AFM by depositing 20 μL of sample on freshly cleaved highly oriented pyrolytic graphite for 30 min followed by a 200‐μL wash with sterile filtered Milli‐Q and drying of the surface with nitrogen gas. The samples were imaged on a Bruker Multimode 8 scanning probe microscope with a Nanoscope V controller, using the ScanAsyst peak‐force tapping imaging mode. Bruker ScanAsyst‐Air probes (nominal spring constant of 0.4 N·m^−1^ and nominal tip radius of 2 nm) were used to scan 5 μm × 5 μm areas of the surface at a resolution of 1024 × 1024 pixels. The images were flattened using Bruker Nanoscope Analysis software to remove tilt and bow. The images were further processed in Matlab and 43 individual fibrils were traced and digitally straightened, which allowed the measurement of peak and through heights from the fibril centre line height profile and the repeat periodicity. Fibril periodicity was determined using the highest frequency peak from the Fourier transform of the fibril centre line height profile. Only filaments with a twisted appearance were selected for analysis.

## Results

**Figure 1:**
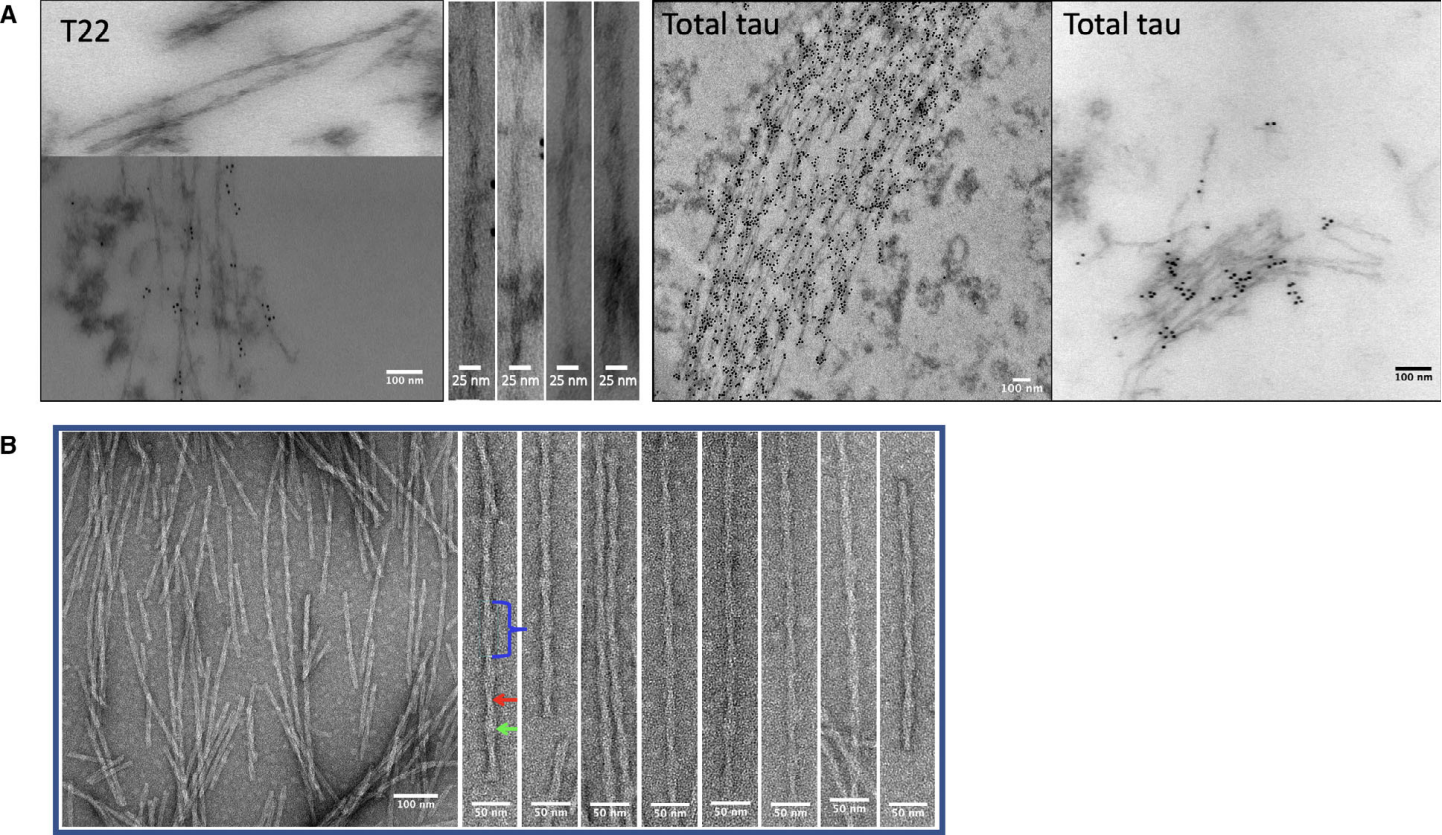
(A) NFTs of tau from AD brain section immunogold labelled with anti‐tau antibody T22 and total tau as labelled in the images. (B) PHFs formed *in vitro* from dGAE. Apparent diameter (mean ± SE) for narrow (Red arrow) and wide (Green arrow) sections of the filaments was 9.47 ± 0.86 nm and 17.42 ± 0.72 nm, respectively. The crossover repeat distance (blue bracket) for all filaments was 72.50 ± 0.94 nm.

**Figure 2:**
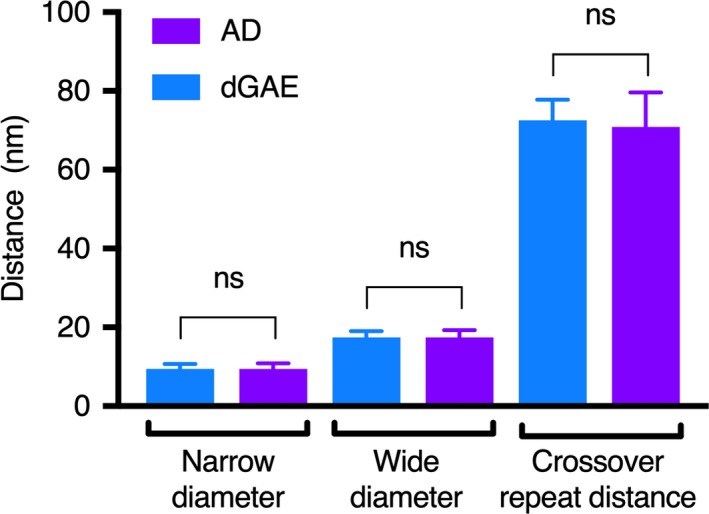
Comparison of twisted dGAE filaments and PHFs from AD brain tissue. Data were analysed using one‐way ANOVA with multiple comparisons and Sidak’s multiple comparisons test. Eighty‐two images were analysed for dGAE using five grids. Forty‐three images were analysed for AD PHFs using three grids from one case. Narrow diameter comparison – mean diff. 0.03291 nm, 95% Cl of diff. −2.047 to 2.113 nm. Not significant (number of measurements – dGAE: 95, AD: 17). Wide diameter comparison – mean diff. −0.2626 nm, 95% Cl of diff. −1.763 to 1.711 nm. Not significant (number of measurements – dGAE: 101, AD: 26). Crossover repeat comparison – mean diff. 1.631 nm, 95% Cl of diff. −0.6283 to 3.890 nm. Not significant (number of measurements – dGAE: 66, AD: 15).

Negatively stained brain sections from an individual diagnosed with AD were examined using TEM in order to visualise the morphology of the filaments *in situ* (in tissue without further processing or extraction) (Fig. 1A). dGAE was incubated with agitation under reducing conditions for 48 h before examination by TEM. The conditions for assembly have been optimised to provide a consistent and even distribution of PHF (with some SFs, approximately 10%) 24. The diameters and periodicity of the PHFs were measured (Fig. 1B) and those formed *in vivo* and *in vitro* were compared. Results revealed no significant variation (*P *> 0.05) within different repeats of dGAE filament preparations and no significant difference between the dGAE and Alzheimer’s PHFs for wide, narrow and crossover repeat measurements (Fig. 2).

**Figure 3:**
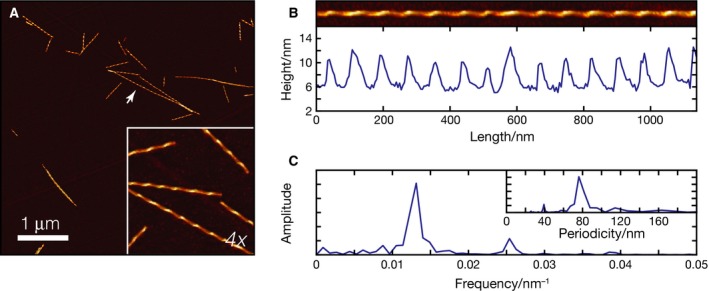
AFM images of filaments made from dGAE under identical conditions as for TEM. (A) A 5 × 5 μm AFM image of dGAE filaments with inset representing a cropped 4× magnified view. (B) Image of a typical traced and digitally straightened fibril (arrow in A) with the height profile of along the centre of the fibril. (C) The Fourier transformed height profile showing peak representing the frequency of the repeating pattern due to fibril twist, with insert representing the periodicity (1/frequency) of the twist. Measurements (mean ± SE) from *n* = 43 filaments are: periodicity, 72.7 ± 1.6 nm; peak height, 11.7 ± 0.2 nm; and trough height, 5.5 ± 0.1 nm.

dGAE filaments prepared in the same way as for TEM were allowed to dry on freshly cleaved highly oriented pyrolytic graphite and subsequent AFM imaging revealed that the filaments formed by dGAE have a left‐handed twist (Fig. 3), the same handedness as reported for AD PHFs 27. Periodicity measurements were indistinguishable from those obtained using TEM, with *in vivo* and *in vitro* filaments, having the same average periodicity of 72.7 ± 1.6 nm (Fig. 3B,C). The average filament centre height measurements for these filaments reveal an average of 11.7 ± 0.2 nm for the peaks and 5.5 ± 0.1 nm for the troughs (Fig. 3B). The AFM height measurements are marginally smaller than the apparent diameters measured from negative stain TEM. This would be expected, since AFM height measurements are likely underestimates of filament width due to compressing effects from the AFM tip, while negative stain TEM diameter measurements are likely overestimates of filament width arising from the apparent widening effect of the negative stain envelope. However, the ratio between AFM peak and trough heights of the dGAE filaments is 2.1 ± 0.3 nm, consistent with the ratio between thick and thin filament regions of 1.8 ± 0.4 nm from TEM images for dGAE filaments. Therefore, all of the imaging data taken together demonstrate that truncated tau forms filaments that share macromolecular characteristics with PHFs found *in vivo.*

**Figure 4:**
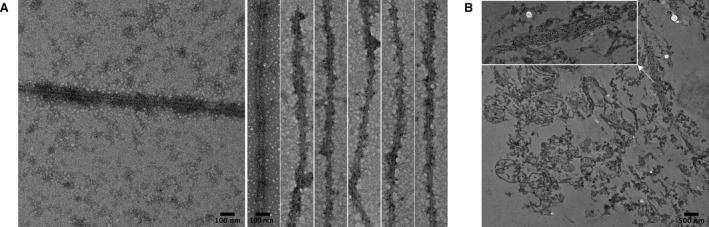
Immunogold labelling TEM with mAb 423 of (A) PHFs formed *in vitro* from dGAE and (B) NFTs of tau from AD brain section.

To further compare the structural characteristics of filaments formed by dGAE with those found in brain tissue, we performed immunogold labelling using an antibody which was raised against proteolytically stable PHF preparations from AD brain tissues and which recognises tau C‐terminally truncated at Glu‐391 19, 26. There was significant antibody labelling of dGAE filaments (Fig. 4A) and immunogold labelling was also observed in AD brain tissue sections (Fig. 4B). This suggests that the regions of tau accessible to this antibody are present in both the dGAE filaments formed *in vitro* and those found *in situ*.

## Discussion

In this study, we report that filaments formed from the truncated tau (297‐391) closely resemble PHFs found in AD brains at the macromolecular level. Our work reveals that, from negative stain images, the *in vitro* filaments share similar characteristics with those observed in AD brain, including width and periodicity. We also reveal that they share similar epitopes exposed on the surface of the filaments.

The relatively low resolution of these results precludes conclusions being drawn regarding the precise molecular interactions within the core structure. Indeed, filaments deposited in chronic traumatic encephalopathy share similar morphology with those from AD brains, whereas cryo‐EM has revealed subtle differences at the molecular level 9. Work is ongoing to characterise the molecular details of the PHFs formed from dGAE, but only cryo‐EM or ssNMR will be able to provide definitive proof regarding the comparison between the synthetic core tau fragment filaments and PHFs formed in AD brain and other conditions. It is not known as yet whether the subtle ultrastructural differences between morphologically similar filaments, forming from the same or homologous underlying tau units in different disease settings, are critical either for understanding the molecular mechanism of assembly of the core tau unit or in the development of novel therapeutic approaches.

Here, the preparation of PHFs from truncated tau was achieved without the addition of heparin. This work also reveals that phosphorylation of tau is not required for the formation of PHFs *in vitro*. Here, we show that a tau fragment can form filaments spontaneously which are similar at the macromolecular level to those deposited in AD brain tissue, and this may provide a tractable model system for understanding the molecular mechanism of assembly of the core domain and for screening inhibitors.

## Author contributions

YA managed the project, conceived the idea and prepared the samples. YA and BF collected and analysed the TEM data; LB provided training and sectioned the brain sample; YA, LL and WFX collected the AFM data; and LL and WFX analysed the AFM data. JER prepared the purified dGAE protein. YA, BF and LS wrote the first draft and all authors reviewed the manuscript and approved the final version. CH, CW and LS managed the overall project.

## Ethics

AD brain tissue was provided by the London Neurodegenerative Diseases Brain Bank, Institute of Psychiatry, King’s College, London, under Local Ethics Committee guidelines, and informed consent for brain donation was obtained from the next of kin by the London Neurodegenerative Diseases Brain Bank.
